# High sensitivity Troponins In Patients with elevated prohormone of beta natriuretic peptide and acute heart failure (HIGH TRIP Trial)

**DOI:** 10.1038/s41598-022-05759-x

**Published:** 2022-02-03

**Authors:** Wesam A. Alhejily

**Affiliations:** 1 Division of Cardiology, Department of Medicine, Faculty of Medicine, King Abdelaziz University Hospital, P.O. Box 80215, Jeddah, 21589 Saudi Arabia; 2 Cardiology Division, Dr Sulaiman Alhabib Medical Center, Riyadh, Saudi Arabia

**Keywords:** Biochemistry, Biomarkers, Cardiology

## Abstract

In patients presented to emergency rooms, Pro hormone of Natriuretic Peptide (Pro BNP) essay is overly sensitive test to rule out heart failure but less specific in predicting outcomes in follow-ups, in this study we ought to find the added value of High Sensitivity cardiac Troponin I (Hs-cTn I), in patients presented acutely with heart failure and its impact on mortality when Pro BNP is highly elevated. Prospective cohort study, inclusion criteria were age above 18 and clearly positive NT Pro BNP > 1000 pg/ml, with 12 months follow up period, primary end point was mortality from heart failure, secondary endpoint was need for rehospitalization. 95 patients were enrolled, divided into overt and non-overt pulmonary edema groups. Mean (Pro BNP) was 6184 and 5927 pg/ml and mean (Hs-cTn I) were 19.27 and 0.17 ng/ml respectively, Mean Ejection fraction was 48 ± 7 and 47 ± 7 for each group sequentially. Mortality rate was 4 (13%) in the higher Hs-c Tn I group, and 1 (1.6%) in the low troponin level group p = .03, odd ratio was 8.5, 95% CI (0.9–80). Need for re-hospitalization was present in 12 (38%) Vs 7 (8%) patients, p = .0081, odd ratio 4.8, 95% CI (1.7–14.2). In COX proportional hazard analysis, only Hs-cTn I was a significant predictor of poor outcome in this high-risk cohort with p = 0.0001. Adding (Hs-cTrop I) assay to the panel of laboratory testing, in patients presented to ER with acute heart failure and with high Pro-BNP > 1000, may further predicts mortality and rehospitalization rate.

## Introduction

Acute cardiac pulmonary edema secondary to heart failure carries a bad prognosis. Beta natriuretic peptide (BNP) and N-terminal Pro BNP are the gold standard biomarkers used for diagnosis and prognosis, more novel biomarkers like Mid-Regional pro ADrenoMedullin (MR-proADM) Mid-Regional pro Atrial Natriuretic Peptide (MR-proANP), Highly sensitive cardiac Troponins (Hs-cTn), soluble Suppression of Tumorigenicity 2 (sST2), Growth Differentiation Factor (GDF)-15 and Galectin-3, may have a role to play in determining prognosis far and beyond the one established by natriuretic peptides, but their potential role in clinical care of patients with heart failure is yet to be fully determined^1^.

There are many studies that looked at the utility of using pro-BNP, the natural peptides produced by atria, due to stretching effect of elevated cavitary pressure, and its value in diagnosing pulmonary edema. It can discriminate cardiac from other causes of lung infiltrates like acute respiratory syndrome and pneumonia. Albeit very well studied^2–6^, BNP and Pro BNP still have some limitations particularly when they are used in follow ups as results of assays may be affected by several factors related to age, gender, body mass, renal function and exercise, all of which may limit its utility for accurately stratifying some patients with acute heart failure^9–14^.

(Hs-cTn) has been suggested to play a role in predicting early onset of heart failure in asymptomatic patients^15,16^ and as a marker of myocardial injury in patients with the first ever presentation of heart failure, based on the Biomarker for Cardiovascular Risk Assessment in Europe consortium (BIOMAReCare) Hs-cTn shows its independence for the prognosis of HF^17–20^.

In this study we ought to find the added value of Hs-cTn in patients presented acutely to the emergency room with dyspnea and highly elevated Pro BNP and its impact on predicting death and re-hospitalization over 12-month time.

## Methods

Cohort prospective study with inclusion criteria of adults above the age of 18, with suspected acute pulmonary edema due to heart failure and elevated -Pro BNP > 1000 pg/ml. Study was approved by the Institutional Review Board (IRB) of Dr Sulaiman Alhabib Medical Group holding approval number HAP-01-R-082, all experiments were performed in accordance with relevant guidelines and regulations and was Health Insurance Portability and Accountability Act (HIPPA) complaint, an informed consent was obtained from all subjects and/or their legal guardian(s). After recruitment, Hs-cTn plasma level was assessed in addition to other laboratory investigations including creatinine, as well as hemoglobin level. All patients underwent chest Xray at presentation. X-rays were read independently by expert radiologists and patient were subclassified into overt pulmonary edema and non-overt pulmonary edema groups based on the reports’ results and were followed for 12 months. Mean of all variances were calculated and analysis of variance (ANOVA) was conducted for non-continuous variables, while (t-test was used for continuous non categorical variables, using 95% power level, looking for p < 0.05 of significance.

**Figure 1:**
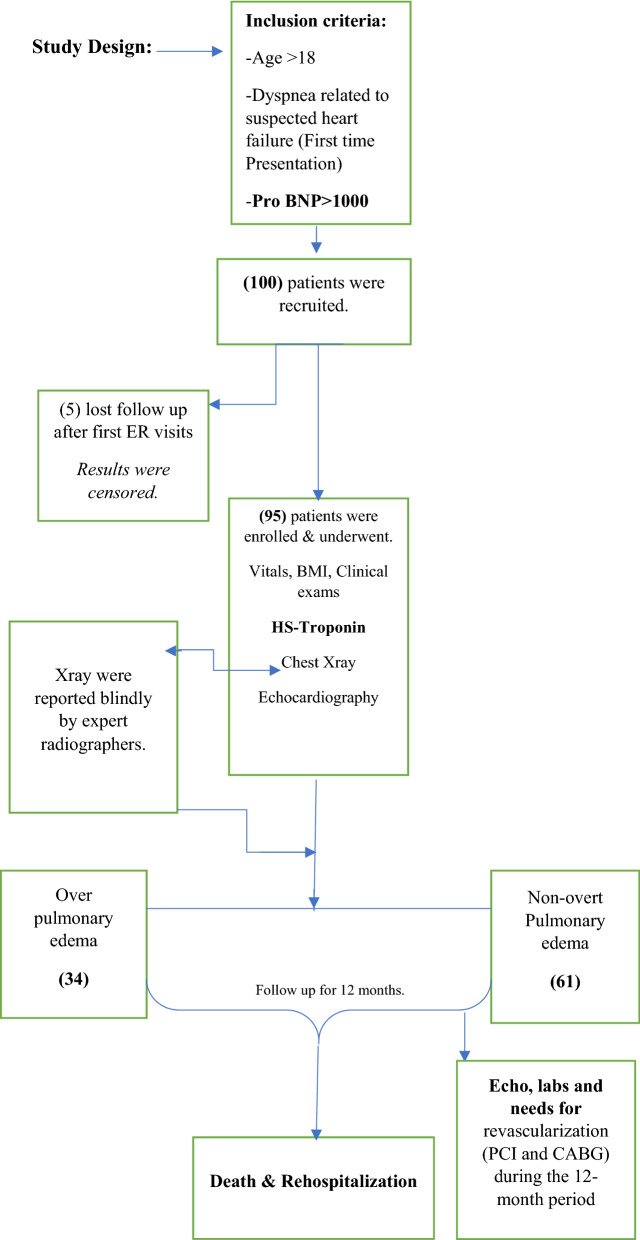
Flowchart of Study Design

During follow up patient’s data were collected including echocardiographic findings, additional special interventions including aggressive medical therapy and revascularization with Percutaneous intervention (PCI) or Bypass surgery (CABG), primary end point was mortality from heart failure and secondary endpoint was need for rehospitalization. Patients who lost follow up were censored and results were not counted in the final analysis. (Fig. 1) explains the study design. Hs-cTns were measured using (Abbott Laboratories; Chicago, IL) with reference range values of (0.004–0.028 ng/ml) and the prohormone brain natriuretic peptide (Pro BNP) was assessed via Elisa immunoassay with 460 pg/ml been the cut value of elevated hormones, we used 1000 pg/ml to avoid borderline positive tests. We did not look at repeated levels of troponins or pro BNP in subsequent presentations as this demonstrated in previous study to be of less predictive value (Fig. 1).

## Results

**Figure 2:**
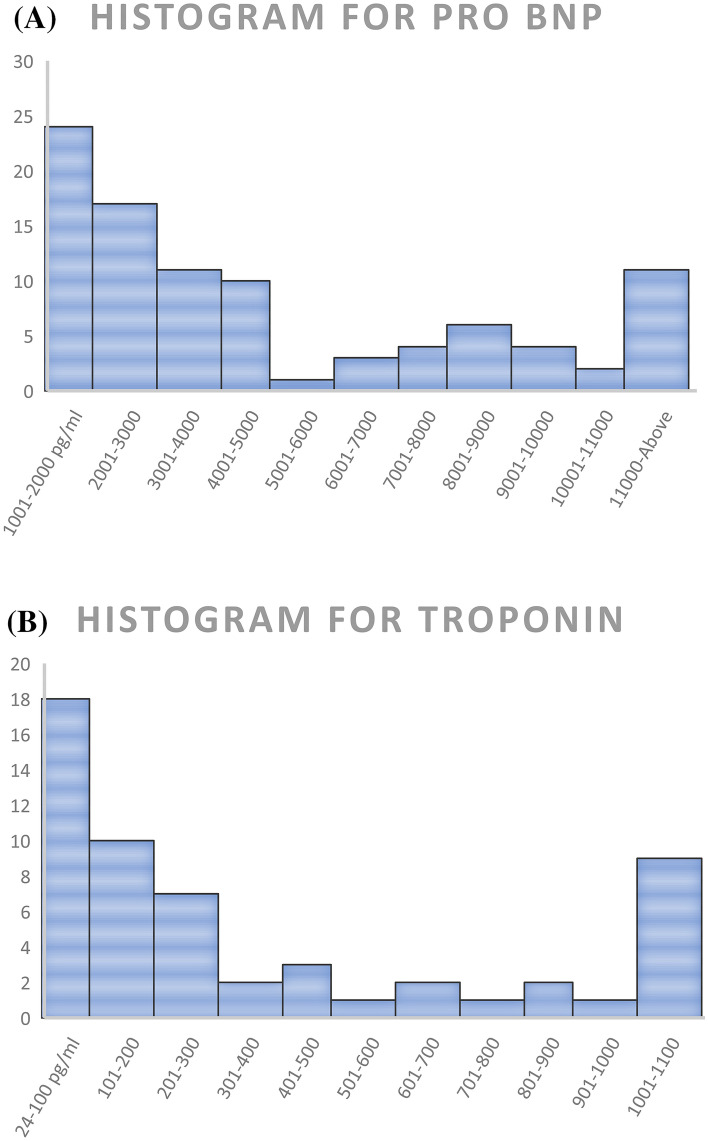
Baseline values of Pro-Bnp and high sensitivity troponin. (**A**) Baseline values of Pro Bnp. X axis represents Pro BNP range values, Y axis represents the number of subjects under each range. (**B**) Baseline values of Hs-CTnI. X axis represents Hs-cTn I range values, Y axis represents the number of subjects under each range.

After 12 months follow up and a mean of 180 days, 95 patients were recruited of which 64 were with non-overt pulmonary edema (non-PE) and 31 with pulmonary edema (PE), mean age was 78 ± 6 and 68 ± 6, males were 51 and 50% on each group. Mean Pro BNP levels were 6148, 5927 pg/ml while Hs-cTn were 19.27 and 0.17 ng/ml respectively (baseline values for both biomarkers are depicted in Fig. 2), mean Body mass index (BMI) of 35 and 29.8, for each group. Other risk factors and clinical findings are depicted on (Table 1), of note there were more cases of atrial fibrillation in the non-overt pulmonary edema group compared to the overt pulmonary edema 41vs 19%. Mean creatinine and hemoglobin blood levels were comparable between both studied groups.

**Table 1:** Patients’ characteristics.

Patient with elevated pro BNP	Overt pulmonary edema N = 34	Non-overt pulmonary edema N = 61
Age	78 ± 6 (SD)	68 ± -6 (SD)
**Gender**		
Male	17 (50%)	31 (51%)
Female	17 (50%)	30 (49%)
**Past medical history**		
DM	24 (71%)	37 (61%)
HTN	3(88%)	46 (75%)
Stroke	2 (3%)	7 (11%)
Chronic lung disease	2(6%)	2 (3%)
Pulmonary embolism	0 (0%)	0 (0%)
Smoking	2 (6%)	2 (3%)
**Physical examination**		
BMI	35.16	29.75
Mean arterial pressure in ER	90.29	90.26
Mean Heart rate	78.64	78.21
Atrial fibrillation on initial ECG	6 (19%)	25 (41%)
**Laboratory findings**		
Creatinine mg/dl	178,49	168.7
Troponin ng/ml	19.27	0.17
HB mg/dl	11.3	11.96
Pro BNP pg/ml	6184.40	5927.76

Echocardiographic findings revealed no significant difference in overall ejection fraction with mean values around 47%, similarly the left atrial size, early diastolic to late diastolic mitral inflow doppler velocity (E/A) ratio, Tricuspid Regurgitation (TR) jet velocity were equivalent between both groups, two exceptions were observed including high Early mitral inflow velocity to early mitral tissue doppler annular velocity E/e’ of 30 compared to 7 in favor of PE group, and the regionality on resting echo was higher among patients in PE group in at least 50% of cases vs only 26% in the non PE (Table 2).

**Table 2:** Echocardiographic findings:

Echocardiographic parameters	Overt pulmonary edema	Non-overt pulmonary edema
Ejection fraction	48.97	47.01
Regionality	17 (50%)	16 (26%)
E	0.97	.89
A	0.71 cm/s	0.62
e′	0.032 cm/s	0.12 cm/s
TR jet velocity	2.34 cm/s	2.84 cm/s
Left atrial size	3.95 cm	4.04 cm
E/A	2.32	2.1
E/eʹ	30	7.4

*E* early mitral inflow doppler velocity, *A* late mitral inflow (Atrial contraction) doppler velocity, *e*ʹ early mitral annular velocity measured by tissue doppler, *TR* tricuspid regurgitation velocity.

**Figure 3:**
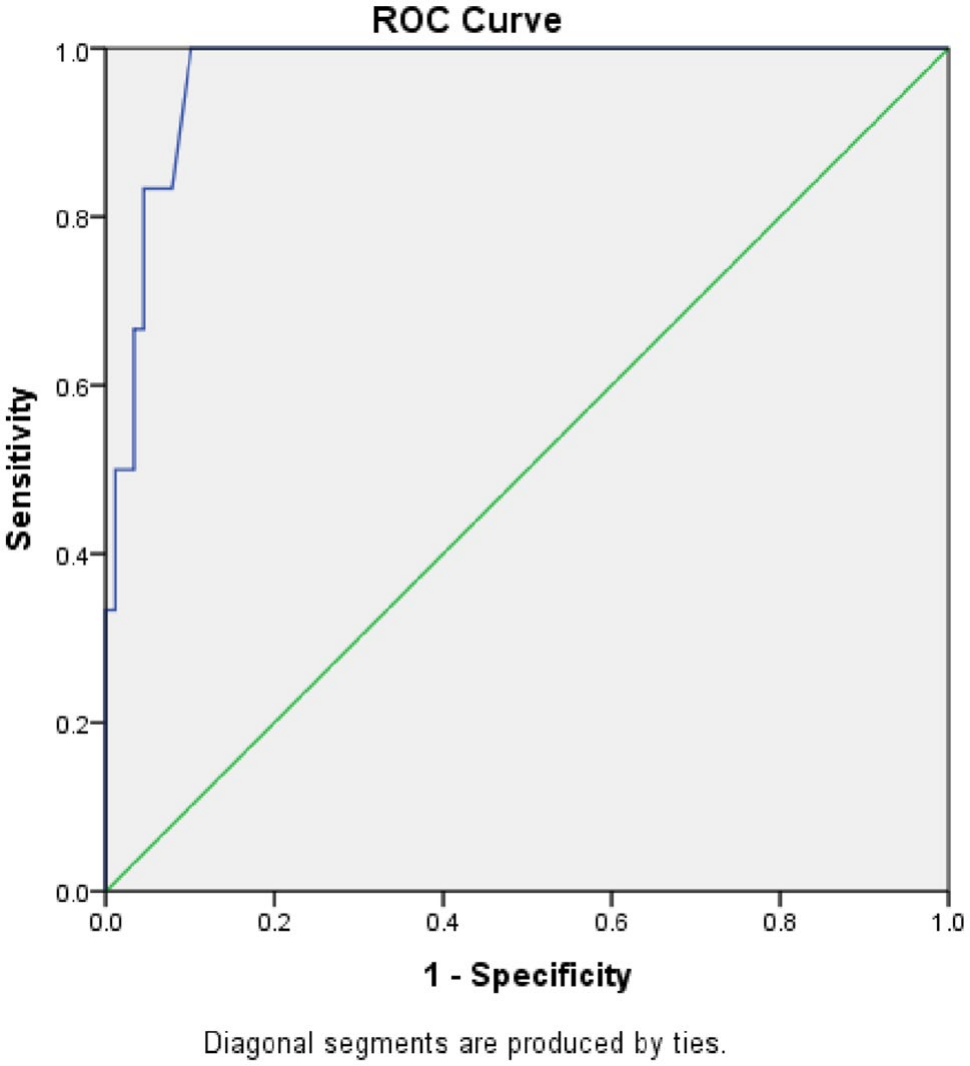
Receiver operator curve for Hs-cTn. X-axis is sensitivity or True positive. Y-axis is 1-specficity or True negative rate. Blue line is test performance with area under the curve of 97%. Green line is when test can’t differentiate true positive from true negative as chances are 50% for both.

**Figure 4:**
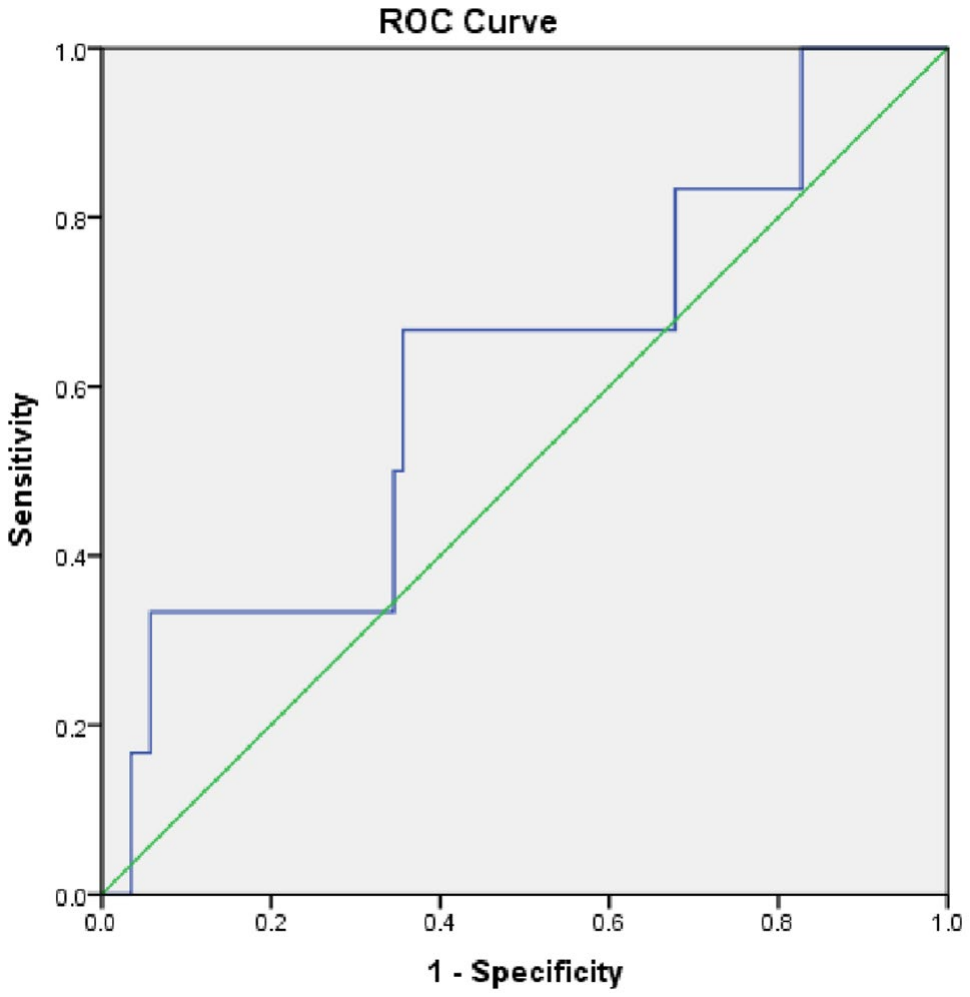
Receiver operator curve of Pro BNP. X-axis is sensitivity or True positive. Y-axis is 1-specficity or True negative rate. Blue line is test performance with area under the curve of 62%. Green line is when test can’t differentiate true positive from true negative as chances are 50% for both.

**Figure 5:**
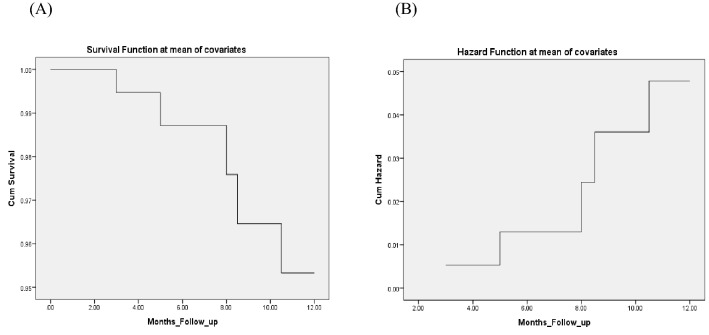
Cox regression curves for cumulative survival and hazard associated with elevated Hs-cTn mean value of 19.27. (**A**) Cumulative survival rate for Hs-Trops in acute heart failure. (**B**) Cumulative hazard rate Hs-Trops in acute heart failure.

For endpoints, we identified 4 mortalities related to heart failure (cardiogenic shock) in the PE compared to 1 in non-PE cohorts, for secondary endpoint 12 cases were hospitalized in the span of 365 days in PE, compared to only 7 in the non-PE. The odd ratios were 8.5 and 4.8 m, p = 0.003 and 0.008 respectively (Table 3), in the COX proportional hazard regression covariate analysis, 7 (covariates) factors were suspected to predict poor outcomes including Pro BNP, Troponin, Creatinine, and diabetes mellitus in addition to E/e’ ratio regionalities and overt pulmonary edema on Xray at the time of presentation , Hs-cTn was the only significant predictor, Hs-cTn is more sensitive and specific compared to Pro BNP in layering the risk of death and accurately predicting poor outcomes, receiver operator curves were obtained for both and depicted on Figs. 3 and 4 with omnibus multivariate logistic regression statistic showed significant p = 0.001, cumulative survival and hazard risk with mean (Hs-cTnI) of 17 has been depicted in Fig. 5.

**Table 3:** Mortality and rehospitalization rate.

Endpoints	Overt pulmonary edema Pro BNP = 6184 Hs-TropI = 19.27	Non overt pulmonary edema Pro BNP = 5927 Hs-Trop I = 0.17	Odd ratio, confidence interval	P value
Mortality in 12 months	4 (13%)	1 (1.6%)	8.5 (0.9–80)	0.03
Rehospitalization in 12 months	12 (38.6%)	7 (11.7)	4.8 (1.7–14.2)	0.0018

Furthermore, need for revascularization was assessed, with 3 vs 7 patients underwent Coronary artery bypass surgery (CABG) and 8 vs 12 patient had Percutaneous Coronary Intervention (PCI) in PE and Non-PE group, respectively. Risk of stroke were similar over all between PE and non-PE groups’ but higher in the subgroup of patient who underwent CABG at 2% compared to overall of 1%. Patients were placed on good medical therapy throughout the study with excellent rate of compliance of more than 85% of cases and were followed in heart function clinic. Patient with reduced ejection fraction < 45% or overt pulmonary edema were treated with diuretics & goal directed medical therapy to reduce mortality and rehospitalization using angiotensin receptor-neprilysin inhibitor (ARNI) or angiotensin blockers with maximum tolerated dose, in addition to beta blockers, aldosterone antagonists and sodium-glucose cotransporter-2 inhibitors. SGLT-2 inhibitors. Those with renal impairment were placed on combination therapy of hydralazine and long-acting nitrate.

## Discussion

Although both were studied before, the added value of Hs-cTn to BNPs is an area that is still under scrutiny^21–23^. The study shed lights on the value of adding one to another in further predicting hard endpoints of heart failure related death and re-hospitalization. There are no “perfect” tests but in this scenario Hs-cTns were “perfect” with sensitivity of 96%. The depicted ROC (receiver operator curves) speaks to this effect very illustratively.

We believe the findings of this trial is unique and may be practice changing for treating patients with acute heart failure. We have selected extremely high-risk patients with quite elevated level of pro Bnp > 1000 and we further stratify them using Hs-cTns and radiological evidence of pulmonary edema, both biomarkers are measured in the laboratories and their blood levels are pathophysiologically explained. BNPs are produced due to stretching effect of the myocardial tissue while high sensitivity troponins are released in blood stream in response to direct damage to the myocardium^24,25^. Translating the laboratories finding to clinical practice was in keeping with the clinical outcomes of patients in this trial, as myocardial damage (measured by high sensitivity Troponins) is more of a surrogate marker compared to the stretching effect causing (Pro BNP elevation).

Despite the novel finding of the trial, there are quite few limitations of the study that need to be highlighted, including small numbers of patients in both groups leading to wide confidence intervals and reducing the power of the study, the selection bias related to open label protocol, and potential lab errors related to sample drawn. The number of patients is low but the events rate over one year, with the clinical characteristics reported, is high, could simply be a chance finding in a small cohort. We used only baseline level of troponin and Pro BNP to assess prognosis without follow-up values, as this has shown to be more predictive of poor outcomes in previous study^26^. We did not repeat cardiac troponin level in post recruitment presentations to avoid dilutional effect of intervention on blood level of pro BNPs and troponins.

The study seems to touch upon major factors of increasing risk of deaths in patients with heart failure including risk factors of ischemic heart disease, reduced ejection fraction, elevated filling pressure by echo criteria and radiological evidence of pulmonary edema, however the study was not powered to look at the impact of all these factors in the small size sample of patients recruited, making it very difficult to compare its utility in addition to Hs-cTns.

Focusing on predicting outcomes is important aspects of proper management of patient with heart failure as outlined in most recent guidelines, where class A is defined as patient at risk of heart failure but not yet diagnosed to have heart failure^27^, adding Hs-cTns to the paradigm of heart failure management may be of value to tailor more aggressive surveillance and more strict management protocol that will help improve survival and outcomes.

The trial served its purpose very well, the drop-out rate was 5%, and results were clinically significant even with the inherited limitations we have alluded to. Further study with large numbers of patients is needed to address the same question but in the setup of other factors or other novel markers, the later may be better in refining the risk assessment tool of patient with first time presentation of heart failure.

## Conclusions

In this small cohort of patient with acute pulmonary edema due to heart failure, high sensitivity cardiac troponins were successful in predicting poor outcomes related to death and hospitalization, Hs-cTn has odd ratio of 8.5 and 4.3 in predicting both respectively despite equal mean level of PRO BNP and ejection fraction.
